# Wigner Functions of Time-Dependent Cat-like Even/Odd Superpositions of Nonlinear Coherent States

**DOI:** 10.3390/e28030287

**Published:** 2026-03-03

**Authors:** Miguel Citeli de Freitas, Viktor V. Dodonov

**Affiliations:** 1 Institute of Physics, University of Brasilia, P.O. Box 04455, Brasilia 70919-970, DF, Brazil; miguelciteli@hotmail.com; 2 International Center for Physics, University of Brasilia, Brasilia 70919-970, DF, Brazil

**Keywords:** coherent states, photon-added coherent states, coherent phase states, nonlinear coherent states, Barut–Girardello coherent states, mid-binomial states, negative binomial states, Pegg–Barnett states, “shark-like” states

## Abstract

We calculate and plot 2D slices of the Wigner functions of several families of highly excited even and odd superpositions of nonlinear coherent states, looking for conditions under which such superpositions can be interpreted as models of the “Schrödinger cat” states. The decisive factor seems to be the form of the number distribution function over the Fock basis: it must have well localized peaks. Otherwise, no “cat-like” structures are observed.

## 1. Introduction

The Wigner function [1] is a useful tool in many areas of quantum mechanics and quantum optics [2,3,4,5,6]. We use the definition (for one degree of freedom and ℏ=1)

(1) $$W\left(q,p\right)=\int_{-\infty }^{\infty }dze^{ipz}\langle q-z/2|\hat{\rho }|q+z/2\rangle ,$$

where $\hat{\rho }$ is a Hermitian positive-definite operator (the statistical operator) normalized as $Tr(\hat{\rho })=1$. In this case, Equation (1) defines a real function with the normalization

(2) $$\int_{-\infty }^{\infty }dq\int_{-\infty }^{\infty }dpW\left(q,p\right)/\left(2\pi \right)=1.$$

It is remarkable that the Wigner function is limited (in contrast to the wave function or the density matrix) [2,7,8,9]:

(3) |W(q,p)|≤2.

The extremal values W(0,0)=±2 are achieved for pure quantum states with definite parity [2,10] whose wave functions obey the relations ψ(x)=±ψ(−x). Moreover, the relation W(q,p)=W(−q,−p) holds for all such states, as can be seen from Equation (1) after the replacement z→−z, writing $\langle x|\hat{\rho }|y\rangle$ as 〈x|ψ〉〈ψ|y〉.

Simple examples of states with definite parity are the Fock states |n〉 of the harmonic oscillator, i.e., eigenstates of the number operator ${\hat{a}}^{†}\hat{a}$, where ${\hat{a}}^{†}$ and $\hat{a}$ are the bosonic creation and annihilation operators satisfying the commutation relation $[\hat{a},{\hat{a}}^{†}]=1$. In this paper, we consider dimensionless canonical position and momentum operators which are related to operators ${\hat{a}}^{†}$ and $\hat{a}$ as follows

(4) $$\hat{q}=\left(\hat{a}+{\hat{a}}^{†}\right)/\sqrt{2},\;\hat{p}=\left(\hat{a}-{\hat{a}}^{†}\right)/\left(i\sqrt{2}\right).$$

The Wigner functions of the Fock states are well known [2,3,4,11,12,13]:

(5) $$W_{n}\left(q,p\right)=2{\left(-1\right)}^{n}\exp\left(-b^{2}\right)L_{n}\left(2b^{2}\right),\;b^{2}=q^{2}+p^{2},$$

where $L_{n}\left(z\right)$ is the Laguerre polynomial defined as [14,15]

$$L_{n}\left(z\right)=\frac{1}{n!}e^{z}\frac{d^{n}}{dz^{n}}\left(e^{-z}z^{n}\right)=\sum_{m=0}^{n}\frac{{\left(-z\right)}^{m}n!}{{\left(m!\right)}^{2}\left(n-m\right)!}.$$

**Figure 1:**
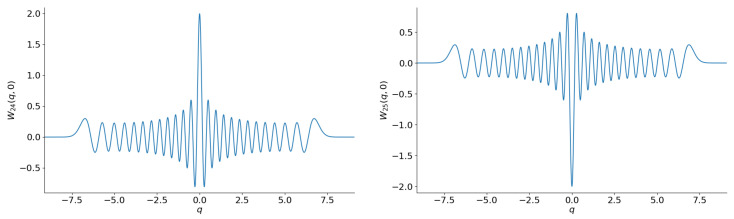
The sections W(q,0) of the Wigner functions of the Fock states with n=24 (**left**) and n=25 (**right**).

Equation (5) strongly oscillate when n≫1 (they have exactly *n* zeros). Consequently, they have approximately *n* circular regions of negativity in the phase plane (q,p), in accordance with the property of the Fock states as being “the most nonclassical states”: see Figure 1.

In addition, these functions are totally symmetric in the phase plane (q,p), since they depend on the only variable $q^{2}+p^{2}$.

On the other hand, both the circular symmetry and oscillating structure totally disappear for the infinite superpositions of the Fock states in the form

(6) $$|\alpha \rangle =\exp\left(-{\left|\alpha \right|}^{2}/2\right)\sum_{n=0}^{\infty }\frac{\alpha^{n}}{\sqrt{n!}}|n\rangle ,\;\hat{a}|\alpha \rangle =\alpha |\alpha \rangle ,$$

known as coherent states (CSs) [16,17,18]. Their Wigner functions have well known Gaussian shapes, centered at the points $\left(\sqrt{2}Re\left(\alpha \right),\sqrt{2}Im\left(\alpha \right)\right)$. The width of these Gaussians does not depend on the complex parameter α. For this reason, coherent states with |α|≫1 are frequently considered as the simplest quantum analogs of macroscopic states.

The states with definite parity can be obtained as the following superpositions of coherent states:

(7) $${|\Psi \rangle }_{\pm ;\alpha }=N_{\pm ;\alpha }\left(|\alpha \rangle \pm |-\alpha \rangle \right),\;N_{\pm ;\alpha }^{-2}=2\left[1\pm \exp\left(-{2|\alpha |}^{2}\right)\right].$$

The states (7) (introduced in paper [19] under the name *even/odd coherent states*) have turned out to be important in many applications for quantum mechanics, quantum optics and quantum information [20,21,22,23,24,25,26,27,28,29]. If |α|≫1, these states can be considered as superpositions of “macroscopically distinguishable quantum states”. For this reason, since the mid-1980s [30,31] and especially the 1990s, many authors have considered them (as well as more general superpositions of coherent or other Gaussian states) as simple models of the famous “Schrödinger cat” states [32,33,34,35,36,37,38,39,40,41,42,43,44,45,46,47]. The names “cat-like states” or “kitten states” are also used, especially for superpositions of more than two coherent states. Various experimental schemes for generating such states have been suggested or realized, e.g., in papers [48,49,50,51,52,53,54,55,56,57,58,59,60,61,62,63,64,65,66,67].

Various generalizations of states (6) and their superpositions have been studied over the past decades [68]. In connection to this, it seems natural to ask whether all such superpositions can be considered as models of “cat-like” states. Searching for an answer to this question, we considered generalizations of states (7) of the form

(8) $${|\Psi \rangle }_{\pm ;\varepsilon }=N_{\pm ;\varepsilon }\left(|\varepsilon \rangle \pm |-\varepsilon \rangle \right),\;N_{\pm ;\varepsilon }^{-2}=2\left(1\pm \langle -\varepsilon |\varepsilon \rangle \right),$$

(9) $$|\varepsilon \rangle =N_{f}\sum_{n=0}^{\infty }\frac{\varepsilon^{n}}{f(n)}|n\rangle ,\;N_{f}^{-2}=\sum_{n=0}^{\infty }\frac{{\left|\varepsilon \right|}^{2n}}{f^{2}\left(n\right)},$$

where f(n) is some positive function and $\varepsilon =\left|\varepsilon \right|e^{i\varphi }$ is a complex number. The choice of the power dependence $\varepsilon^{n}$ in the definition (9) is not accidental: due to the equidistant energy spectrum of the harmonic oscillator, the time evolution of the state (9) is reduced to the substitution $\varepsilon \to \varepsilon e^{-it}$ (in the dimensionless units with ω=1). Hence, the variations in phase φ are equivalent to the temporal evolution of the quantum state (9). In view of this observation, we plot the sections W(q,0) everywhere for different values of the phase φ. Note that the choice φ=π/2 can also be interpreted as the transformation to the momentum representation: writing the Wigner function in the more detailed form as W(q,p;φ), one can see that W(q,0;π/2)=W(0,p;0).

The states (9) are known under the name “nonlinear coherent states” [69,70,71,72] (although sometimes in slightly different forms). In principle, they can be generated using various schemes of “quantum state engineering” and were elaborated upon, e.g., in papers [73,74,75,76,77]. Even and odd superpositions of nonlinear coherent states were considered, e.g., in papers [78,79,80,81,82,83,84,85,86,87]. It seems interesting to study how functions f(n) different from $\sqrt{n!}$ influence the shape of the corresponding Wigner functions. Earlier, the Wigner functions of the even/odd superpositions of different quantum states were plotted in papers [88,89,90,91], as well as in many of the papers cited above. However, the authors of these studies considered small values of parameters like |ε| or |α| when the mean numbers of quanta in the superpositions were low (frequently, less than 1), due to technical difficulties in numeric calculations with high values of |ε| or |α|. On the other hand, the well known examples of the “usual” even/odd coherent states show that the most interesting behavior can be observed in superpositions with high mean numbers of quanta.

Using the program *Python*, we have succeeded in overcoming the numeric challenges and have plotted the Wigner functions for highly excited superpositions of different families of states like (9). Numerous examples demonstrated that the “cat-like” structures were maintained for rapidly growing (faster than $\sqrt{n!}$) functions f(n). On the other hand, choosing f(n)≡1, we discovered a total disappearance of the “cat-like” structure. Therefore, we investigated several families of well known nonlinear coherent states with different functions f(n), trying to find conditions under which the “cat-like” structures could be observed.

Our plan is as follows: In Section 2, we recount the well known properties of the usual even/odd coherent states and clarify our vision of the “cat-like” structures. The remarkable family of “photon-added” states admitting exact analytical expressions for the Wigner functions is considered in Section 3. In Section 4, we state general formulas for arbitrary functions f(n). In Section 5, we show examples of superpositions with rapidly growing functions $f_{g}\left(n\right)=\sqrt{n!\Gamma (n+g+1)}$ (known under the name “Barut–Girardello coherent states”), which maintain the “cat-like” structure. Interesting special cases of “binomial states” are demonstrated in Section 6. Superpositions of “coherent phase states” with f(n)=1 are considered in Section 7. Plots of the Wigner functions for more general “negative binomial states” with $f_{\nu }\left(n\right)={\left[n!/\Gamma (n+\nu )\right]}^{1/2}$ and different values of parameter ν are shown in Section 8. Examples of the even/odd states with wide flat number distribution functions are considered in Section 9. Section 10 contains our conclusions.

## 2. Wigner Functions of the Usual Even/Odd Coherent States

The wave function of the usual coherent state (6) in the coordinate representation has the well known Gaussian form

(10) $$\langle x|\alpha \rangle =\pi^{-1/4}\exp\left(-\frac{1}{2}x^{2}+\sqrt{2}x\alpha -\frac{1}{2}\alpha^{2}-\frac{1}{2}{\left|\alpha \right|}^{2}\right).$$

In this case, the right-hand side of Equation (1) for the states (7) is reduced to the calculation of four simple Gaussian integrals according to the decomposition of the statistical operator

$$\hat{\rho }=N_{\pm ;\alpha }^{2}\left(|\alpha \rangle \langle \alpha |+|-\alpha \rangle \langle -\alpha |\pm |\alpha \rangle \langle -\alpha |\pm |-\alpha \rangle \langle \alpha |\right).$$

The result can be expressed as follows

(11) $$W_{\pm ;\alpha }\left(q,p\right)={\left[1\pm e^{-{2|\alpha |}^{2}}\right]}^{-1}\left(e^{-|\zeta -\sqrt{2}{\alpha |}^{2}}+e^{-|\zeta +\sqrt{2}{\alpha |}^{2}}\pm 2e^{-{\left|\zeta \right|}^{2}}\cos\left[2\sqrt{2}Im\left(\zeta \alpha^{*}\right)\right]\right),$$

where $\alpha =\left|\alpha \right|e^{i\varphi }$ and $\zeta =q+ip=be^{i\chi }$ with $b=\sqrt{q^{2}+p^{2}}$.

Function (11) describes two symmetrical Gaussian peaks of an approximate unit width, centered at the points $\pm \sqrt{2}\alpha$ in the complex ζ plane, together with the central interference peak, whose height (or depth) is twice of the height of the lateral peaks. The shape of this peak is strongly asymmetric: it is smooth Gaussian in the direction of the lateral peaks, whereas strong oscillations can be observed in the perpendicular direction. The width of the envelope of the central peak is the same as that of the lateral peaks. All three peaks are well separated if |α|≫1.

**Figure 2:**
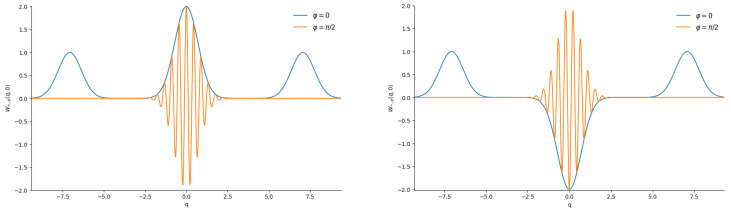
The sections W(q,0) of the Wigner functions of the even (**left**) and odd (**right**) coherent states with |α|=5 and two extreme phases: φ=0 and φ=π/2.

One can see that the right-hand side of Equation (11) depends on the difference in phases Φ=φ−χ between complex numbers α and ζ. Therefore, the shape of the Wigner function is preserved if the variation in phase φ is compensated by the same variation in phase χ. In terms of the temporal evolution, this means that the initial Wigner function uniformly rotates along the axis perpendicular to the (q,p) plane without any deformation. Many authors illustrated the shape of the Wigner function using 3D plots. However, although such plots seem attractive at first glance, they have several disadvantages. For example, looking at such plots, it is frequently difficult to extract exact numerical values or to distinguish between regions of positive and negative values of the Wigner function. Moreover, making such plots requires great numerical efforts. We prefer to illustrate the Wigner function with the aid of the usual 2D plots, fixing the phase χ=0 and showing section W(q,0)=W(−q,0) of the total Wigner function for different values of the phase φ of the complex parameter α (or parameter ε in the subsequent sections). Figure 2 shows the Wigner functions $W_{\pm ;\alpha }\left(q,0\right)$ for |α|=5 (when the mean number of quanta in each component equals ${\left|\alpha \right|}^{2}=25$) and two extreme phases: φ=0 and φ=π/2 (the second choice is equivalent to showing the section W(0,p) at the initial instant). Note that lateral peaks do not appear in function W(q,0) for the phase φ=π/2 because they are totally suppressed by the factor $\exp\left(-{2|\alpha |}^{2}\right)$ when |α|≫1. A similar suppression happens in all other examples considered in the following sections.

The number distribution function $p_{n}={\left|\langle \psi |n\rangle \right|}^{2}$ over the Fock states |n〉 has the following expressions for the even/odd coherent states:

(12) $$p_{+;2k}=\frac{{\left|\alpha \right|}^{4k}}{\cosh(|\alpha {|}^{2})\left(2k\right)!},\;p_{-;2k+1}=\frac{{\left|\alpha \right|}^{4k+2}}{\sinh(|\alpha {|}^{2})\left(2k+1\right)!}.$$

**Figure 3:**
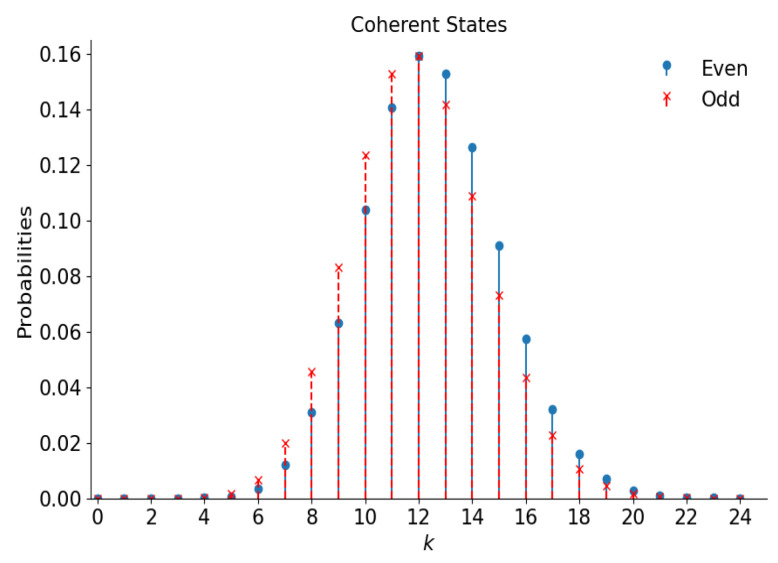
The number distribution functions of the even and odd coherent states with |α|=5.

Plots of these distributions as functions of argument k=0,1,2,… are shown in Figure 3 for |α|=5. The label “probability” at the vertical axis in this figure (and other similar figures) means either $p_{+;2k}$ or $p_{-;2k+1}$, distinguished by different colors and shapes of points. It is worth remembering that even distributions are “super-Poissonian”, whereas odd distributions are “sub-Poissonian” (12).

Note that the maximal probabilities in the even and odd distributions practically coincide. Perhaps this is a mere accidental coincidence. However, it is interesting that the same coincidences can be seen in almost all similar figures, except for the case of “mid-binomial” distributions in Figure 8.

Now we can explain the meaning of the term “cat-like states” accepted in this paper. Namely, in the following sections, we look for the even/odd superpositions whose 2D plots of the section W(q,0) with different phases φ of complex parameters α or ε resemble Figure 2: three well separated peaks for φ=0 and a single strongly oscillating narrow peak at the center for φ=π/2.

## 3. Photon-Added Even/Odd Coherent States

Explicit expressions for the Wigner function were obtained in paper [92] for the “photon-added” even/odd coherent states

(13) $${\left({\hat{a}}^{†}\right)}^{m}\left(|\alpha \rangle \pm |-\alpha \rangle \right),$$

which generalize the photon-added coherent states introduced in paper [93]. (The special case of even/odd states with m=1 was studied in Ref. [94].) The normalized Wigner function of state (13) can be written as follows

(14) $$\begin{array}{ccc} W_{m}^{\pm }\left(q,p\right) & = & \Lambda_{m}^{\pm }\left\{e^{-|\zeta -\sqrt{2}{\alpha |}^{2}}L_{m}\left(|\sqrt{2}{\zeta -\alpha |}^{2}\right)+e^{-|\zeta +\sqrt{2}{\alpha |}^{2}}L_{m}\left(|\sqrt{2}{\zeta +\alpha |}^{2}\right)\right.\\ & & \left.\pm 2e^{-{\left|\zeta \right|}^{2}}Re\left[e^{-2\sqrt{2}iIm\left(\zeta \alpha^{*}\right)}L_{m}\left({2|\zeta |}^{2}-{\left|\alpha \right|}^{2}+2\sqrt{2}iIm\left(\zeta \alpha^{*}\right)\right)\right]\right\}, \end{array}$$

where

(15) $$\Lambda_{m}^{\pm }={\left(-1\right)}^{m}{\left[L_{m}\left(-{\left|\alpha \right|}^{2}\right)\pm e^{-{2|\alpha |}^{2}}L_{m}\left({\left|\alpha \right|}^{2}\right)\right]}^{-1}.$$

Since $|L_{m}\left(x\right)|<e^{x/2}$ for x>0 [14], we have $\Lambda_{m}^{+}\approx \Lambda_{m}^{-}\approx {\left(-1\right)}^{m}{\left[L_{m}\left(-{\left|\alpha \right|}^{2}\right)\right]}^{-1}$ if |α|≫1.

The 3D plots of function (14) were presented in paper [92] for |α|≤1 and m≤10. We have succeeded in making 2D plots for higher values of |α| and *m*. The centers of two lateral peaks seem to be near the points $\zeta =\pm \sqrt{2}\alpha$. Writing $\zeta =\sqrt{2}\alpha +\rho$, we obtain the following expression for the shape of the right peak:

(16) $$W_{m}^{\pm }\left(\rho \right)\approx {\left(-1\right)}^{m}e^{-{\left|\rho \right|}^{2}}\frac{L_{m}\left(|\alpha +\sqrt{2}{\rho |}^{2}\right)}{L_{m}\left(-{\left|\alpha \right|}^{2}\right)}.$$

**Figure 4:**
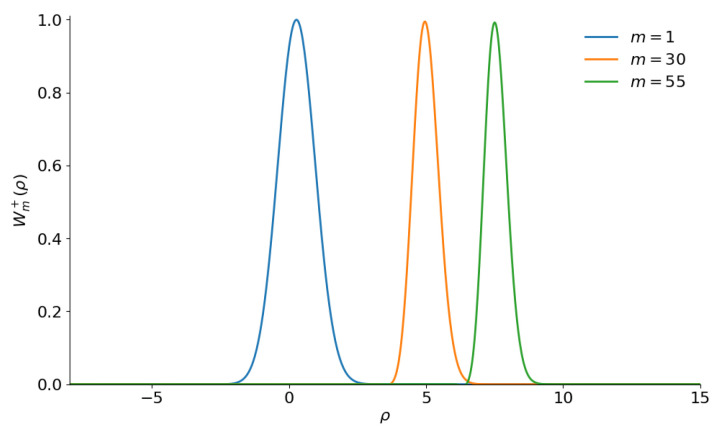
The shape $W_{m}^{+}\left(\rho \right)$ of the Wigner function lateral right peak of the photon-added even coherent states with |α|=5 and different values of parameter *m*.

Remembering that the Laguerre polynomials of positive argument oscillate, one could suppose that function (16) shows some “fine structure” if |α|≫1 and m≫|α|. However, Figure 4 demonstrates quite a different picture: lateral peaks practically maintain their bell shapes without visible oscillations, while their centers move further from the point (0,0) in the q−p plane. Moreover, numerical calculations give the same heights of the peaks $W_{max}=1$ for any value of *m* (as well as for the usual even/odd coherent states with m=0). Note also that the lateral peaks of the even and odd superpositions coincide. The Wigner functions only show the mirror symmetry between the even and odd superpositions for the interference peaks in the region |ζ|≪|α|. However, in this region, we have $L_{m}\left({2|\zeta |}^{2}-{\left|\alpha \right|}^{2}+2\sqrt{2}iIm\left(\zeta \alpha^{*}\right)\right)\approx L_{m}\left(-{\left|\alpha \right|}^{2}\right)$, i.e., the same function as in the denominator of the normalization factor $\Lambda_{m}^{\pm }$. This means that the central interference peak does not depend on integer *m*, except for a possible sign inversion due to the factor ${\left(-1\right)}^{m}$.

**Figure 5:**
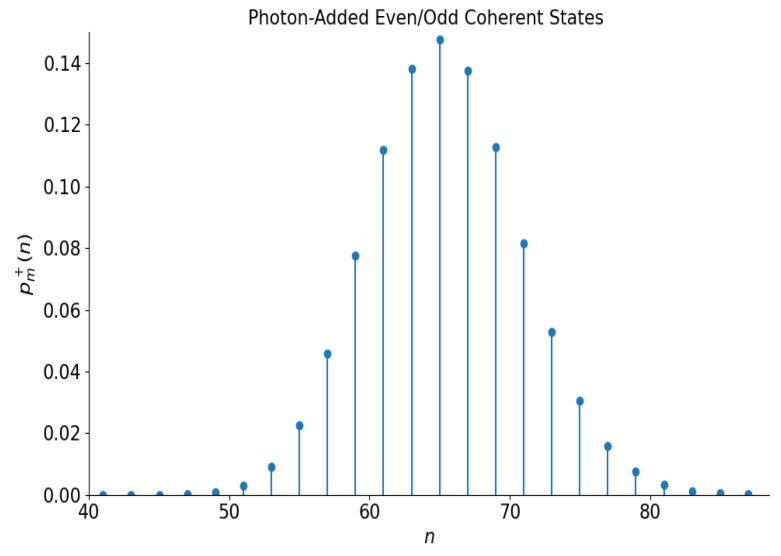
The number distribution function of the even photon-added coherent states with |α|=5 and m=25.

The number distribution function equals

(17) $$p_{m}^{\pm }\left(n\right)=\frac{{n!|\alpha |}^{2(n-m)}e^{-{\left|\alpha \right|}^{2}}\left[1\pm {\left(-1\right)}^{n-m}\right]}{m!{\left[\left(n-m\right)!\right]}^{2}\left[L_{m}\left(-{\left|\alpha \right|}^{2}\right)\pm e^{-{2|\alpha |}^{2}}L_{m}\left({\left|\alpha \right|}^{2}\right)\right]}\;if\;\;n\geq m,$$

and $p_{m}^{\pm }\left(n\right)=0$ if n<m. This distribution is illustrated in Figure 5 for the even superposition with |α|=5 and m=25 (the odd distribution is quite similar). It looks like a shifted distribution of Figure 3.

## 4. General Even/Odd Superpositions

If a pure state |ψ〉 is a superposition of the Fock states,

(18) $$|\psi \rangle =\sum_{k=0}^{\infty }c_{k}|k\rangle ,$$

the corresponding statistical operator ${\hat{\rho }}_{\psi }=\left|\psi \rangle \langle \psi \right|$ can be written as a double sum over the diadic operators |l〉〈r|:

(19) $${\hat{\rho }}_{\psi }=\sum_{l,r=0}^{\infty }c_{l}c_{r}^{*}|l\rangle \langle r|.$$

The related Wigner function can be written as

(20) $$W_{\psi }\left(q,p\right)=\sum_{l,r=0}^{\infty }c_{l}c_{r}^{*}W_{lr}\left(q,p\right),$$

where $W_{lr}\left(q,p\right)$ is the Weyl symbol [95] of the operator |l〉〈r|. This symbol was calculated, e.g., in papers [11,12,13]:

(21) $$W_{lr}\left(q,p\right)=2{\left(-1\right)}^{\mu_{lr}}e^{i\chi (r-l)}L\left(2b^{2};\mu_{lr},\left|l-r\right|\right),\;\mu_{lr}=min\left(l,r\right),$$

(22) $$L\left(z;n,k\right)=\sqrt{\frac{n!}{(n+k)!}}e^{-z/2}z^{k/2}L_{n}^{\left(k\right)}\left(z\right).$$

Here, $L_{n}^{\left(k\right)}\left(z\right)$ is the generalized Laguerre polynomial [14,15],

$$L_{n}^{\left(k\right)}\left(z\right)=\frac{1}{n!}e^{z}z^{-k}\frac{d^{n}}{dz^{n}}\left(e^{-z}z^{n+k}\right)=\sum_{m=0}^{n}\frac{{\left(-z\right)}^{m}\left(n+k\right)!}{m!(m+k)!(n-m)!}.$$

Note that the difference l−r is an even number, both for the even and odd superpositions, whereas $\mu_{lr}$ is an even number for even superpositions and an odd number for odd superpositions. Expansions of superpositions (8) over the Fock basis have the form

(23) $${|\Psi \rangle }_{+;f}=N_{+f}\sum_{n=0}^{\infty }\frac{\varepsilon^{2n}}{f(2n)}|2n\rangle ,$$

(24) $${|\Psi \rangle }_{-;f}=N_{-f}\sum_{n=0}^{\infty }\frac{\varepsilon^{2n+1}}{f(2n+1)}|2n+1\rangle ,$$

(25) $$N_{+f}^{-2}=\sum_{n=0}^{\infty }\frac{{\left|\varepsilon \right|}^{4n}}{f^{2}\left(2n\right)},\;N_{-f}^{-2}=\sum_{n=0}^{\infty }\frac{{\left|\varepsilon \right|}^{4n+2}}{f^{2}\left(2n+1\right)}.$$

The total Wigner function (20) of even (odd) states (23) or (24) can be written as $W_{1}+W_{2}$, where the first series $W_{1}$ contains the terms with m=n only (hence, it does not depend on phases φ and χ), whereas the summation in the double series $W_{2}$ over all numbers l≠r can be performed with respect to independent coefficients λ=|n−m|=1,2,… and μ=0,1,2,…. The explicit expressions are as follows

(26) $$W_{+1;f}\left(q,0\right)=2N_{+f}^{2}\;e^{-q^{2}}\sum_{n=0}^{\infty }\frac{{\left|\varepsilon \right|}^{4n}}{{\left[f\left(2n\right)\right]}^{2}}L_{2n}\left(2q^{2}\right),$$

(27) $$W_{-1;f}\left(q,0\right)=-2N_{-f}^{2}\;e^{-q^{2}}\sum_{n=0}^{\infty }\frac{{\left|\varepsilon \right|}^{4n+2}}{{\left[f\left(2n+1\right)\right]}^{2}}L_{2n+1}\left(2q^{2}\right),$$

(28) $$W_{+2;f}\left(q,0\right)=4N_{+f}^{2}\sum_{\mu =0}^{\infty }\sum_{\lambda =1}^{\infty }\frac{{\left|\varepsilon \right|}^{2(2\mu +\lambda )}\cos\left(2\lambda \varphi \right)}{f(2\mu )f(2\mu +2\lambda )}L\left(2q^{2};2\mu ,2\lambda \right),$$

(29) $$W_{-2;f}\left(q,0\right)=-4N_{-f}^{2}\sum_{\mu =0}^{\infty }\sum_{\lambda =1}^{\infty }\frac{{\left|\varepsilon \right|}^{2(2\mu +\lambda +1)}\cos\left(2\lambda \varphi \right)}{f(2\mu +1)f(2\mu +2\lambda +1)}L\left(2q^{2};2\mu +1,2\lambda \right).$$

In the numeric calculations performed in the following sections, we truncated the series at some indexes like $n_{max}$, chosen in such a way that further increases in this number practically did not change the result. In particular, all series in Section 7 and Section 8 were calculated up to the maximal summation indexes $n_{max}=\mu_{max}=\lambda_{max}=80$.

## 5. Even/Odd “Barut–Girardello” Coherent States

As an example of rapidly converging superpositions of the Fock states (faster than in the standard coherent states), we considered the so-called “Barut–Girardello coherent states” [96] (also studied in [19]) with the nonlinearity function

(30) $$f\left(n\right)=\sqrt{n!\Gamma (n+g+1)},$$

where Γ(z) is the Euler gamma-function and *g* arbitrary real parameter. The normalization constants (25) can be expressed in terms of the Bessel functions of the first and third kinds (in this section, we use the letter γ instead of α and ε):

(31) $$2N_{\pm ;\gamma }^{-2}={\left|\gamma \right|}^{-g}\left[I_{g}\left(2|\gamma |\right)\pm J_{g}\left(2|\gamma |\right)\right].$$

**Figure 6:**
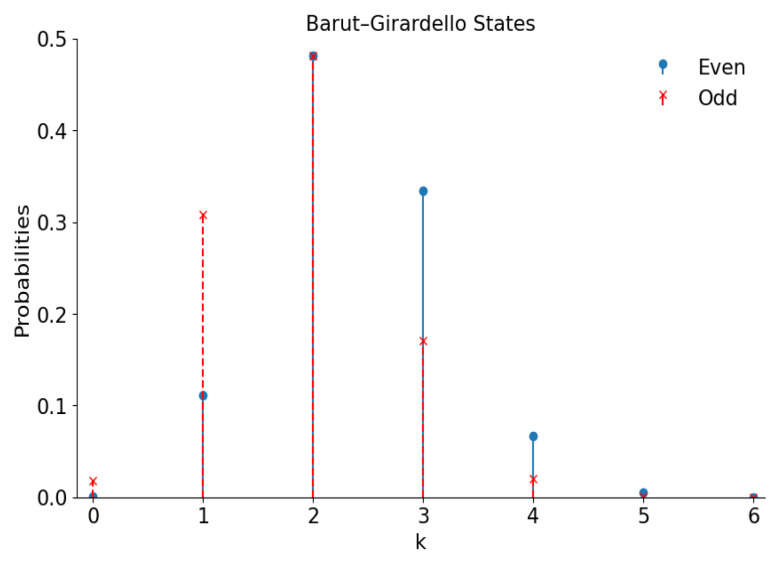
The number distribution functions of the even and odd superpositions of the Barut–Girardello coherent states with |γ|=5.

Figure 6 shows the number distributions in the even/odd superpositions of the Barut–Girardello coherent states with g=0 and |γ|=5,

(32) $$p_{+;2k}=\frac{{2|\gamma |}^{4k}}{\left[I_{0}\left(2|\gamma |\right)+J_{0}\left(2|\gamma |\right)\right]{\left[\left(2k\right)!\right]}^{2}},\;p_{-;2k+1}=\frac{{2|\gamma |}^{4k+2}}{\left[I_{0}\left(2|\gamma |\right)-J_{0}\left(2|\gamma |\right)\right]{\left[\left(2k+1\right)!\right]}^{2}}.$$

**Figure 7:**
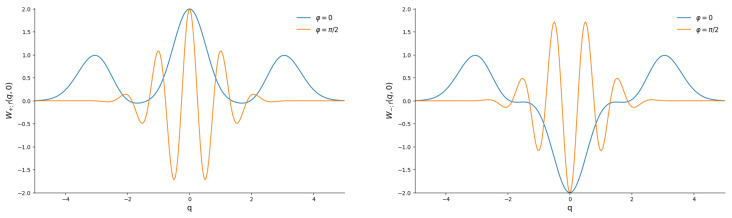
The sections W(q,0) of the Wigner functions of the even (**left**) and odd (**right**) Barut–Girardello coherent states with function f(n) given by Equation (30); for g=0, |γ|=5 and there are two phases.

Figure 7 shows practically the same “cat” structures of the Wigner functions as in Figure 2.

## 6. Superpositions of Binomial States

We consider normalized binomial states in the form

(33) $$|\varepsilon ;M\rangle ={\left(1+{\left|\varepsilon \right|}^{2}\right)}^{-M/2}\sum_{n=0}^{M}{\left[\frac{M!}{n!(M-n)!}\right]}^{1/2}\varepsilon^{n}|n\rangle .$$

Such states (or their modifications and generalizations) were considered, e.g., in Refs. [97,98,99,100]. The mean number of quanta and its variance in state (33) are as follows

(34) $${\langle n\rangle }_{M}=\frac{{M|\varepsilon |}^{2}}{1+{\left|\varepsilon \right|}^{2}},\;\sigma_{n}^{\left(M\right)}=\frac{{M|\varepsilon |}^{2}}{{\left(1+{\left|\varepsilon \right|}^{2}\right)}^{2}}.$$

Defining the nonlinearity function as

(35) $$f\left(n\right)={\left[\frac{M!}{n!(M-n)!}\right]}^{-1/2}$$

and using Newton’s binomial expansion, one can find the normalization factors (25) of the even/odd superpositions:

(36) $$N_{\pm M}^{-2}=\frac{1}{2}\left[{\left(1+{\left|\varepsilon \right|}^{2}\right)}^{M}\pm {\left(1-{\left|\varepsilon \right|}^{2}\right)}^{M}\right].$$

Phase-dependent parts of the Wigner functions are finite double sums:

$$W_{+2;M}\left(q,0\right)=\sum_{\mu =0}^{[\frac{M}{2}]-1}\sum_{\lambda =1}^{[\frac{M}{2}]-\mu }\frac{4N_{+M}^{2}e^{-q^{2}}M!\;{\left|\varepsilon \right|}^{2(2\mu +\lambda )}\cos\left(2\lambda \varphi \right){\left(2q^{2}\right)}^{\lambda }L_{2\mu }^{2\lambda }\left(2q^{2}\right)}{\left(2\mu +2\lambda \right)!\sqrt{(M-2\mu )!(M-2\mu -2\lambda )!}},$$

$$W_{-2;M}\left(q,0\right)=-\sum_{\mu =0}^{\left[\frac{M-3}{2}\right]}\sum_{\lambda =1}^{[\frac{M-1}{2}]-\mu }\frac{4N_{-M}^{2}e^{-q^{2}}M!\;{\left|\varepsilon \right|}^{2(2\mu +\lambda +1)}\cos\left(2\lambda \varphi \right){\left(2q^{2}\right)}^{\lambda }L_{2\mu +1}^{2\lambda }\left(2q^{2}\right)}{\left(2\mu +2\lambda +1\right)!\sqrt{(M-2\mu -1)!(M-2\mu -2\lambda -1)!}}.$$

The phase-independent parts of the Wigner functions $W_{\pm 1;M}\left(q,0\right)$ can be calculated analytically with the aid of Formula (4.4.1.7) from Ref. [101]

(37) $$\sum_{n=0}^{M}\frac{M!}{n!(M-n)!}z^{n}L_{n}\left(x\right)={\left(1+z\right)}^{M}L_{M}\left(\frac{zx}{1+z}\right).$$

The results are as follows

$$W_{\pm 1;M}\left(q,0\right)=N_{\pm ,M}^{2}\;e^{-q^{2}}\left[{\left(1-{\left|\varepsilon \right|}^{2}\right)}^{M}L_{M}\left(\frac{2q^{2}{\left|\varepsilon \right|}^{2}}{{\left|\varepsilon \right|}^{2}-1}\right)\pm {\left(1+{\left|\varepsilon \right|}^{2}\right)}^{M}L_{M}\left(\frac{2q^{2}{\left|\varepsilon \right|}^{2}}{1+{\left|\varepsilon \right|}^{2}}\right)\right].$$

These expressions can be simplified for the “mid-binomial” states with ${\left|\varepsilon \right|}^{2}=1$ (when ${\langle n\rangle }_{M}=M/2$, $\sigma_{n}^{\left(M\right)}=M/4$ and $N_{+M}^{2}=N_{-M}^{2}=2^{1-M}$):

(38) $$W_{\pm 1;M}^{mid}\left(q,0\right)=\pm 2e^{-q^{2}}\left[L_{M}\left(q^{2}\right)\pm q^{2M}/M!\right].$$

The probability distributions over the Fock states have the following forms for “mid-binomial” states with |ε|=1:

(39) $$p_{+;2k}=\frac{M!2^{1-M}}{(2k)!(M-2k)!},\;p_{-;2k+1}=\frac{M!2^{1-M}}{(2k+1)!(M-2k-1)!}.$$

**Figure 8:**
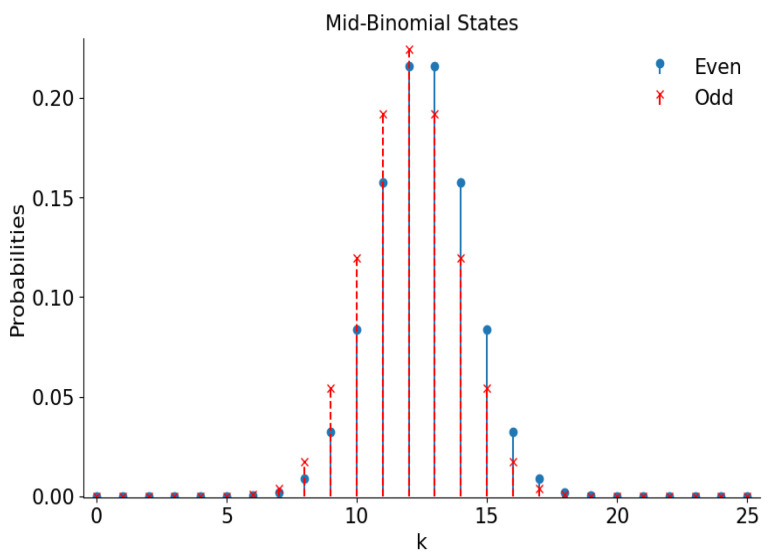
The number distribution functions of the even and odd superpositions of the “mid-binomial” states with M=50.

These distributions are shown in Figure 8 for M=50.

**Figure 9:**
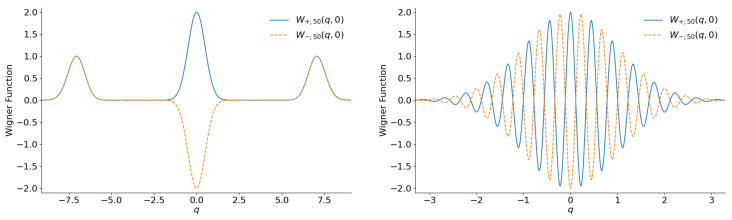
The sections W(q,0) of the Wigner functions of the even and odd superpositions of “mid-binomial” states with M=50. (**Left**): For φ=0. (**Right**): For φ=π/2.

Plots of the Wigner functions of the even/odd superpositions of the “mid-binomial” states with M=50 are shown in Figure 9. They look very similar to the plots for usual even/odd coherent states in Figure 2.

## 7. Even/Odd Coherent Phase States

Among an infinite number of possible functions f(n) in Equation (9), the simplest choice seems to be f(n)≡1. It produces the family of *coherent phase states* (CPSs [102,103,104,105,106,107,108,109,110,111] (also called “harmonious” [112], “pseudothermal” [113] and “geometric” [114] states):

(40) $$|\varepsilon \rangle =\sqrt{1-{\left|\varepsilon \right|}^{2}}\sum_{n=0}^{\infty }\varepsilon^{n}|n\rangle ,\;\varepsilon =\left|\varepsilon \right|e^{i\varphi },\;\left|\varepsilon \right|<1.$$

Even and odd superpositions of the CPSs have the following normalized expansions in the Fock basis:

(41) $${|\Psi \rangle }_{+;\varepsilon }=\sqrt{1-{\left|\varepsilon \right|}^{4}}\sum_{n=0}^{\infty }\varepsilon^{2n}|2n\rangle ,$$

(42) $${|\Psi \rangle }_{-;\varepsilon }={\left|\varepsilon \right|}^{-1}\sqrt{1-{\left|\varepsilon \right|}^{4}}\sum_{n=0}^{\infty }\varepsilon^{2n+1}|2n+1\rangle .$$

**Figure 10:**
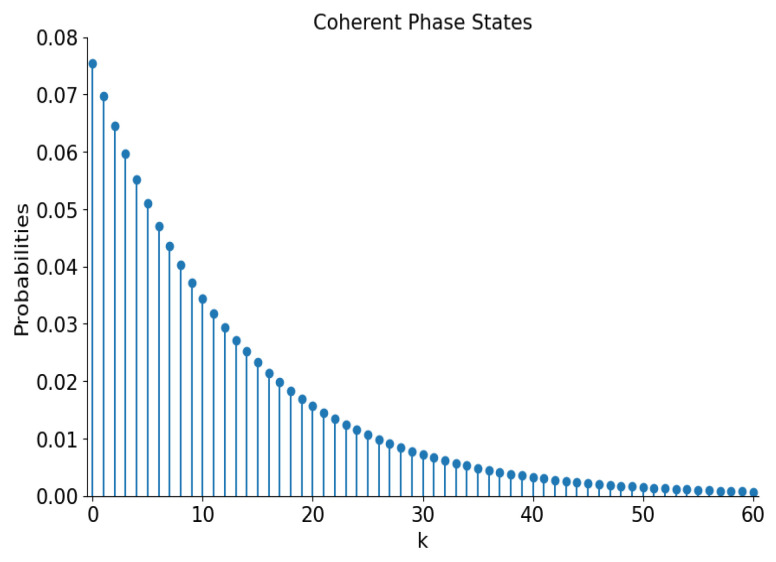
The number distribution functions of the even and odd superpositions of the coherent phase states with ${\left|\varepsilon \right|}^{2}=25/26$.

In this case, we have thermal-like distributions (slowly and monotonously decaying),

(43) $$p_{+;2k}=\left(1-{\left|\varepsilon \right|}^{4}\right){\left|\varepsilon \right|}^{4k}=p_{-;2k+1},$$

which are shown in Figure 10 for ${\left|\varepsilon \right|}^{2}=25/26$.

The superpositions (41) and (42) were considered in paper [115]. However, the corresponding Wigner functions were plotted there for small values of parameter |ε| only, while the most interesting features could be observed in the limit |ε|→1, as we demonstrate below.

A detailed study of the statistical properties of the even/odd coherent phase states (squeezing, the uncertainty products, Mandel’s factor, etc.) was performed recently in paper [116]. Here, we show the plots of the corresponding Wigner functions. In fact, their total difference from the case of usual even/odd coherent states induced us to write the present paper.

The series (26) and (27) with f(n)=1 can be calculated analytically as the even and odd parts $\left[S_{x}\left(z\right)\pm S_{x}\left(-z\right)\right]$ of the known generating function for the Laguerre polynomials,

(44) $$S_{x}\left(z\right)=\sum_{n=0}^{\infty }L_{n}\left(x\right)z^{n}={\left(1-z\right)}^{-1}\exp\left(\frac{xz}{z-1}\right).$$

The results are as follows

(45) $$W_{+1;\varepsilon }\left(q,0\right)=\left(1-{\left|\varepsilon \right|}^{2}\right)\exp\left(-q^{2}\frac{1-{\left|\varepsilon \right|}^{2}}{1+{\left|\varepsilon \right|}^{2}}\right)+\left(1+{\left|\varepsilon \right|}^{2}\right)\exp\left(-q^{2}\frac{1+{\left|\varepsilon \right|}^{2}}{1-{\left|\varepsilon \right|}^{2}}\right),$$

(46) $$W_{-1;\varepsilon }\left(q,0\right)={\left|\varepsilon \right|}^{-2}\left[\left(1-{\left|\varepsilon \right|}^{2}\right)\exp\left(-q^{2}\frac{1-{\left|\varepsilon \right|}^{2}}{1+{\left|\varepsilon \right|}^{2}}\right)-\left(1+{\left|\varepsilon \right|}^{2}\right)\exp\left(-q^{2}\frac{1+{\left|\varepsilon \right|}^{2}}{1-{\left|\varepsilon \right|}^{2}}\right)\right].$$

One can see that $W_{\pm ;\varepsilon }\left(0,0\right)=\pm 2$, as it must be for even or odd states. The off-diagonal parts of the Wigner functions can be reduced to the following double series:

(47) $$W_{+2;\varepsilon }\left(q,0\right)=4\left(1-{\left|\varepsilon \right|}^{4}\right)\sum_{\mu =0}^{\infty }\sum_{\lambda =1}^{\infty }{\left|\varepsilon \right|}^{2(2\mu +\lambda )}\cos\left(2\lambda \varphi \right)L\left(2q^{2};2\mu ,2\lambda \right),$$

(48) $$W_{-2;\varepsilon }\left(q,0\right)=-4\left(1-{\left|\varepsilon \right|}^{4}\right)\sum_{\mu =0}^{\infty }\sum_{\lambda =1}^{\infty }{\left|\varepsilon \right|}^{2(2\mu +\lambda )}\cos\left(2\lambda \varphi \right)L\left(2q^{2};2\mu +1,2\lambda \right).$$

Unfortunately, these series can be calculated only numerically.

**Figure 11:**
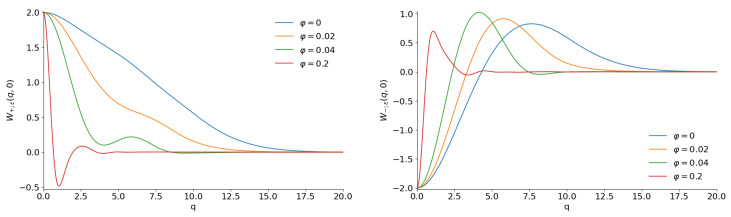
The sections W(q,0) of the Wigner functions of the even (**left**) and odd (**right**) coherent phase states with ${\left|\varepsilon \right|}^{2}=25/26$ and different phases.

**Figure 12:**
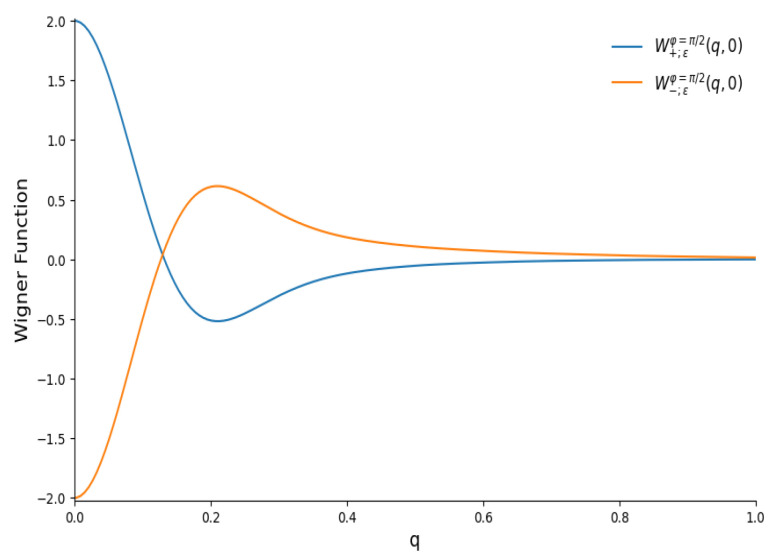
The section W(q,0) of the Wigner functions of the even and odd coherent phase states with ${\left|\varepsilon \right|}^{2}=25/26$ and φ=π/2.

Figure 11 shows the Wigner functions $W_{\pm ;\varepsilon }\left(q,0\right)$ for ${\left|\varepsilon \right|}^{2}=25/26$ (when the mean number of quanta in each component equals ${\langle n\rangle }_{CPS}={\left|\varepsilon \right|}^{2}{/(1-|\varepsilon |}^{2})=25$) and different phases. We plot the figures for q>0 in view of the relation W(q,0)=W(−q,0). One can see a strong difference between Figure 2 and Figure 11. For example, there are no lateral peaks for φ=0 in Figure 11. A probable explanation is the strong connection between the variance $\sigma_{x}$ and the mean value 〈x〉 in the coherent phase states with φ=0 [111]: $\sigma_{x}\approx 0.4\langle n\rangle$ and $\langle x\rangle \approx 1.25\sqrt{\langle n\rangle }$. Therefore, the peaks of two components of the even superposition of CPSs merge into a single central peak, in contrast to the even superpositions of the usual CSs, whose peak widths do not depend on 〈x〉 and 〈n〉. Although something remotely similar to lateral peaks is visible for odd superpositions, these peaks are smooth continuations of the central peak, whereas lateral peaks in usual odd CSs are well separated from the central peak. Another striking difference from the case of usual even/odd coherent states is the absence of any “fine structure” for the phase φ=π/2, as can be seen in Figure 12.

## 8. Even/Odd Negative Binomial States

Coherent phase states can be considered as a special case (ν=1) of *negative binomial states* (NBSs), whose normalized expansion over the Fock states has the form

(49) $$|\varepsilon ,\nu \rangle ={\left(1-{\left|\varepsilon \right|}^{2}\right)}^{\nu /2}\sum_{n=0}^{\infty }{\left[\frac{\Gamma (n+\nu )}{\Gamma (\nu )n!}\right]}^{1/2}\varepsilon^{n}|n\rangle ,$$

with simple expressions for the mean number of quanta and its variance [114]:

(50) $${\langle n\rangle }_{\nu }=\frac{{\nu |\varepsilon |}^{2}}{1-{\left|\varepsilon \right|}^{2}},\;\sigma_{n}^{\left(\nu \right)}=\frac{{\nu |\varepsilon |}^{2}}{{\left(1-{\left|\varepsilon \right|}^{2}\right)}^{2}}.$$

The early history of studies of such states occurred in papers [19,103,114,117,118,119,120,121]. Even and odd NBSs were considered in papers [122,123]. The normalization factor in Equation (49) follows from Formula (5.2.11.16) in [124]

(51) $$\sum_{n=0}^{\infty }\frac{\Gamma (n+\nu )}{\Gamma (\nu )n!}z^{n}={\left(1-z\right)}^{-\nu }.$$

Consequently, using the function

(52) $$f\left(n\right)={\left[\frac{n!}{\Gamma (n+\nu )}\right]}^{1/2},$$

we obtain the following normalization coefficients in Equation (25):

(53) $$N_{\pm ,\nu }^{2}=\frac{2}{\Gamma (\nu )}{\left[{\left(1-{\left|\varepsilon \right|}^{2}\right)}^{-\nu }\pm {\left(1+{\left|\varepsilon \right|}^{2}\right)}^{-\nu }\right]}^{-1}.$$

The number distributions are as follows

(54) $$p_{+;2k}=\frac{{2|\varepsilon |}^{4k}\Gamma \left(2k+\nu \right)}{\Gamma \left(\nu \right)\left[{\left(1-{\left|\varepsilon \right|}^{2}\right)}^{-\nu }+{\left(1+{\left|\varepsilon \right|}^{2}\right)}^{-\nu }\right]\left(2k\right)!},$$

(55) $$p_{-;2k+1}=\frac{{2|\varepsilon |}^{4k+2}\Gamma \left(2k+1+\nu \right)}{\Gamma \left(\nu \right)\left[{\left(1-{\left|\varepsilon \right|}^{2}\right)}^{-\nu }-{\left(1+{\left|\varepsilon \right|}^{2}\right)}^{-\nu }\right]\left(2k+1\right)!}.$$

To plot the Wigner functions, we calculate the following series numerically:

(56) $$W_{+1;\nu }\left(q,0\right)=2N_{+,\nu }^{2}\;e^{-q^{2}}\sum_{n=0}^{\infty }\frac{\Gamma (2n+\nu )}{(2n)!}{\left|\varepsilon \right|}^{4n}L_{2n}\left(2q^{2}\right),$$

(57) $$W_{-1;\nu }\left(q,0\right)=-2N_{-,\nu }^{2}\;e^{-q^{2}}\sum_{n=0}^{\infty }\frac{\Gamma (2n+1+\nu )}{(2n+1)!}{\left|\varepsilon \right|}^{4n+2}L_{2n+1}\left(2q^{2}\right),$$

(58) $$\begin{array}{ccc} & & W_{+2;\nu }\left(q,0\right)=4N_{+,\nu }^{2}e^{-q^{2}}\sum_{\mu =0}^{\infty }\sum_{\lambda =1}^{\infty }{\left|\varepsilon \right|}^{2(2\mu +\lambda )}{\left(2q^{2}\right)}^{\lambda }\\ & & \times \frac{\sqrt{\Gamma (2\mu +\nu )\Gamma (2\mu +2\lambda +\nu )}}{(2\mu +2\lambda )!}\cos\left(2\lambda \varphi \right)L_{2\mu }^{\left(2\lambda \right)}\left(2q^{2}\right), \end{array}$$

(59) $$\begin{array}{ccc} & & W_{-2;\nu }\left(q,0\right)=-4N_{-,\nu }^{2}e^{-q^{2}}\sum_{\mu =0}^{\infty }\sum_{\lambda =1}^{\infty }{\left|\varepsilon \right|}^{2(2\mu +\lambda +1)}{\left(2q^{2}\right)}^{\lambda }\\ & & \times \frac{\sqrt{\Gamma (2\mu +1+\nu )\Gamma (2\mu +2\lambda +1+\nu )}}{(2\mu +2\lambda +1)!}\cos\left(2\lambda \varphi \right)L_{2\mu +1}^{\left(2\lambda \right)}\left(2q^{2}\right). \end{array}$$

**Figure 13:**
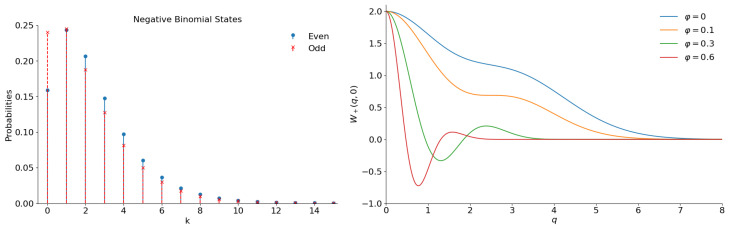
Illustrations for the even and odd superpositions of the negative binomial states with ${\left|\varepsilon \right|}^{2}=5/7$ and ν=2. (**Left**): The number distribution functions. (**Right**): Function W(q,0) for the even superpositions with different values of phase φ.

Numeric calculations for the values ν≠1 result in figures looking qualitatively like Figure 11 and Figure 12, provided the difference |ν−1| is not very big. Figure 13 illustrates the case of ν=2.

**Figure 14:**
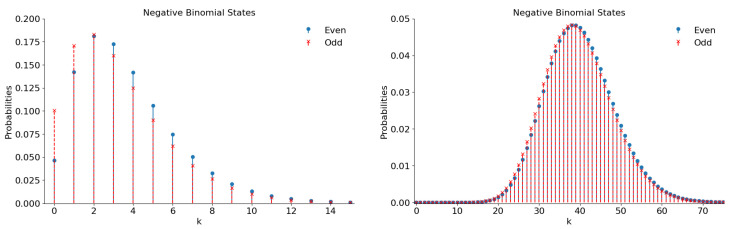
The number distribution functions of the even and odd superpositions of the negative binomial states with ${\left|\varepsilon \right|}^{2}=5/7$ for ν=3 (**left**) and ν=32 (**right**).

If parameter ν increases, the number distribution function acquires more and more pronounced peaks; see Figure 14. The related plots for two different values of parameter ν are shown in Figure 14.

Note that Equation (50) yields the mean number of quanta in each component of the superposition 〈n〉=80 for ν=32 and ${\left|\varepsilon \right|}^{2}=5/7$.

**Figure 15:**
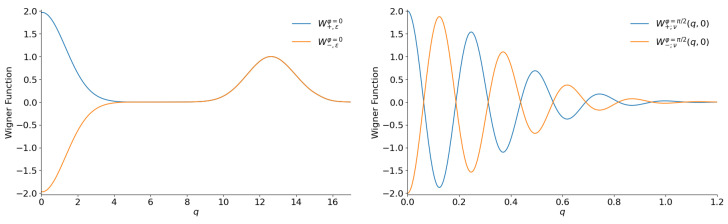
The section W(q,0) of the Wigner functions of the even and odd negative binomial states with ν=32 and ${\left|\varepsilon \right|}^{2}=5/7$ for φ=0 (**left**) and φ=π/2 (**right**).

Finally, the “cat” structure is totally revived for ν≫1, as can be seen in Figure 15.

In the limit case of ν→0, the only surviving term in the even number distribution (54) is $p_{+;0}^{\left(\nu =0\right)}=1$ for any value of |ε|. This means that even superpositions of the NBS go to the vacuum state |0〉 in the limit ν→0. The Wigner function of this state has a well known Gaussian form, which is positive everywhere. Plots of the Wigner functions of even superpositions (not given here) show only small negative regions for ν<1, which are practically invisible for ν≪1.

For the odd superpositions of NBSs, using the formula $\Gamma \left(\nu \right)\to \nu^{-1}$ for ν→0, we arrive at the monotonous number distribution, which decays faster than for odd coherent phase states:

(60) $$p_{-;2k+1}^{\left(\nu =0\right)}=\frac{{2|\varepsilon |}^{4k+2}}{\ln\left(\frac{1+{\left|\varepsilon \right|}^{2}}{1-{\left|\varepsilon \right|}^{2}}\right)\left(2k+1\right)}.$$

The components of the Wigner function assume the following forms:

(61) $$W_{-1;0}\left(q,0\right)=-\frac{4e^{-q^{2}}}{\ln\left(\frac{1+{\left|\varepsilon \right|}^{2}}{1-{\left|\varepsilon \right|}^{2}}\right)}\sum_{n=0}^{\infty }\frac{{\left|\varepsilon \right|}^{4n+2}}{2n+1}L_{2n+1}\left(2q^{2}\right),$$

(62) $$\begin{array}{ccc} W_{-2;0}\left(q,0\right) & = & -\frac{8e^{-q^{2}}}{\ln\left(\frac{1+{\left|\varepsilon \right|}^{2}}{1-{\left|\varepsilon \right|}^{2}}\right)}\sum_{\mu =0}^{\infty }\sum_{\lambda =1}^{\infty }{\left|\varepsilon \right|}^{2(2\mu +\lambda +1)}{\left(2q^{2}\right)}^{\lambda }\\ & & \times \frac{\sqrt{(2\mu )!(2\mu +2\lambda )!}}{(2\mu +2\lambda +1)!}\cos\left(2\lambda \varphi \right)L_{2\mu +1}^{\left(2\lambda \right)}\left(2q^{2}\right). \end{array}$$

**Figure 16:**
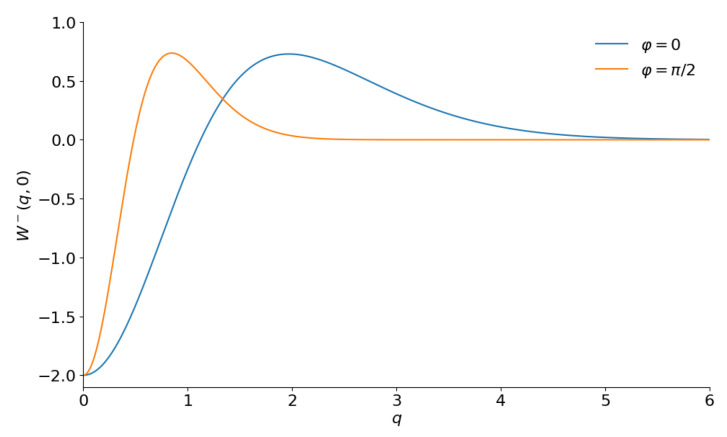
The sections W(q,0) of the Wigner functions of the odd negative binomial states with ν=0 and ${\left|\varepsilon \right|}^{2}=5/7$ for φ=0 and φ=π/2.

The corresponding slices of the Wigner function for ${\left|\varepsilon \right|}^{2}=5/7$ are shown in Figure 16.

They look similar to those in Figure 11 and Figure 12 for ν=1 (i.e., for the odd coherent phase states).

## 9. Even/Odd Superpositions with Flat Number Distribution Functions

Comparing the results of the preceding sections, we thought (as the first hypothesis) that the key factor in generating ”cat-like” states could be the form of the number distribution function over the Fock basis p(n). Namely, it seems that no “cat” structures can appear if function p(n) decreases monotonously. Indeed, such a behavior was observed for the negative binomial states with ν≤1, according to Section 7 and Section 8. On the other hand, if function p(n) [more precisely, p(2k) and p(2k+1)] increases initially and then decays, the “cat” structures are possible, as was shown in Section 2, Section 3, Section 5, Section 6 and Section 8 for ν≫1.

To check this initial hypothesis, we considered several examples of finite even/odd superpositions of (K+1)≫1 Fock states with flat number distribution functions of the following form:

(63) $$|\psi ;KM+\rangle =\frac{1}{\sqrt{K+1}}\sum_{k=M}^{M+K}e^{2ik\varphi }|2k\rangle ,\;|\psi ;KM-\rangle =\frac{1}{\sqrt{K+1}}\sum_{k=M}^{M+K}e^{(2k+1)i\varphi }|2k+1\rangle .$$

If M=0, these are even and odd superpositions of the Pegg–Barnett truncated phase states [125]. Now, the Wigner function can be written in terms of finite single and double sums:

(64) $$W_{+1}^{KM}\left(q,0\right)=\frac{2e^{-q^{2}}}{K+1}\sum_{n=M}^{M+K}L_{2n}\left(2q^{2}\right),$$

(65) $$W_{-1}^{KM}\left(q,0\right)=-\frac{2e^{-q^{2}}}{K+1}\sum_{n=M}^{M+K}L_{2n+1}\left(2q^{2}\right),$$

(66) $$W_{+2}^{KM}\left(q,0\right)=\frac{4e^{-q^{2}}}{K+1}\sum_{\mu =M}^{M+K-1}\sum_{\lambda =1}^{M+K-\mu }\cos\left(2\lambda \varphi \right){\left(2q^{2}\right)}^{\lambda }\sqrt{\frac{(2\mu )!}{(2\mu +2\lambda )!}}L_{2\mu }^{\left(2\lambda \right)}\left(2q^{2}\right).$$

(67) $$W_{-2}^{KM}\left(q,0\right)=-\frac{4e^{-q^{2}}}{K+1}\sum_{\mu =M}^{M+K-1}\sum_{\lambda =1}^{M+K-\mu }\cos\left(2\lambda \varphi \right){\left(2q^{2}\right)}^{\lambda }\sqrt{\frac{(2\mu +1)!}{(2\mu +2\lambda +1)!}}L_{2\mu +1}^{\left(2\lambda \right)}\left(2q^{2}\right).$$

**Figure 17:**
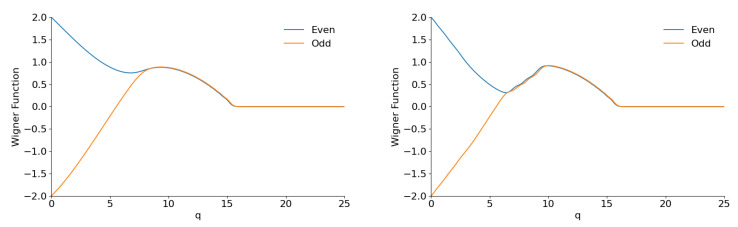
The section W(q,0) of the Wigner functions of the even/odd flat superpositions with φ=0 and K=60 for M=0 (**left**) and M=3 (**right**).

**Figure 18:**
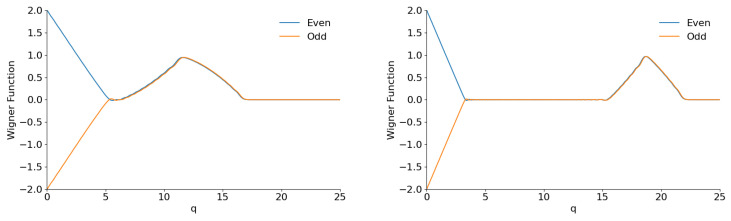
The section W(q,0) of the Wigner functions of the even/odd flat superpositions with φ=0 and K=60 for M=10 (**left**) and M=60 (**right**).

Examples of the Wigner function sections with φ=0 are shown in Figure 17 and Figure 18.

We see nothing similar to Figure 2 in Figure 17 for small values of parameter *M*. When this parameter increases, the lateral peak moves further from the central one, and it becomes totally separated for M≫1, as shown in Figure 18. However, both peaks do not have the bell shape typical for “cat” states: their forms are close to triangles. The name “shark-like” states is probably more adequate for superpositions illustrated in Figure 18. The plots in this figure, especially for M=60, can look strange, because they seem to be made from pieces of straight lines. Indeed, all analytical expressions for the Wigner function contain $q^{2}$ in their arguments. This means that W(q,0) must have a parabolic form for q→0, as one can see in Figure 2 (and in other similar figures). In fact, precise calculations confirm initial parabolic behavior, but it happens in a very small region, which is not seen in the scale used in Figure 18. The same can be said about the transitions to the zero values of the Wigner functions. They seem to be very “sharp”, but we have checked and these transitions are smooth, although the transition intervals are very small.

**Figure 19:**
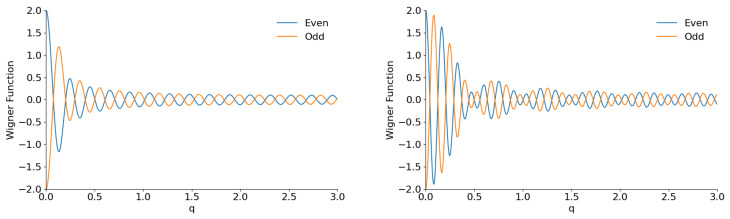
The section W(q,0) of the Wigner functions of the even/odd flat superpositions with K=150 and φ=π/2 for M=0 (**left**) and M=25 (**right**).

Another difference between the “cat” and “shark” states can be seen in Figure 19 for φ=π/2. The plots show fast oscillations with almost constant amplitude until very big values of coordinate *q* occur. These plots are totally different not only from Figure 2 for usual even/odd coherent states but also from Figure 12 for the even/odd coherent phase states.

## 10. Conclusions

The main achievement of this paper is that we have succeeded in plotting 2D sections of the Wigner functions of highly excited even/odd superpositions of several families of nonlinear coherent states with high values of parameters, thus improving results of numerous studies performed by other authors during the several preceding decades. The plots demonstrated in this paper show that the family of possible “cat-like” quantum states is significantly wider than was thought earlier. One of decisive factors seems to be the form of the number distribution function over the Fock basis p(n). Several different examples show that “cat-like” states with bell-shape slices of the Wigner function W(q,0) can be observed if function p(n) is non-monotonous, having pronounced peaks (containing many Fock states). The “shark-like” functions W(q,0) with long oscillating “tails” in W(0,p) are observed for “flat” distribution functions with constant nonzero values in the intervals M≤n≤M+K if M≫1 and K≫1. No “cat-like” structures are observed for monotonously decreasing functions p(n) starting from n=0, as well as for “flat” distributions beginning from small numbers *M*. It is interesting that the heights of lateral peaks of the function W(q,0) with φ=0 are exactly twice as small as the height of the central interference peak in all examples where the “cat-like” or “shark-like” structures are clearly seen (whereas these heights are significantly smaller when such structures do not appear, like in Figure 16). We can suppose that more complicated Wigner functions describing “macroscopic quantum superpositions” can be observed for even/odd superpositions of nonlinear coherent states possessing more sophisticated nonlinearity functions f(n). But this can be a subject of other studies. Another new direction for future studies is related to attempts to understand how the shapes of new “cat-like” states can influence the decoherence, “classicalization” and thermalization times of different initial “macroscopic” superpositions in the presence of different reservoirs (comparing, e.g., with the results of studies [23,90,126,127,128,129,130]).

## Data Availability

No new data were created or analyzed in this study. Data sharing is not applicable to this article.
